# Supplementary material for: Characterization of the Barley Net Blotch Pathosystem at the Center of Origin of Host and Pathogen
Source: Pathogens. 2019 Nov 29;8(4):275. doi: 10.3390/pathogens8040275 (PMC6963742; doi:10.3390/pathogens8040275)
Supplement: Supplementary file 1 [file pathogens-08-00275-s001.zip › pathogens-649293-supplementary/supplementary.docx]

**Table S1.** Distribution of Net Blotch disease in Israel by site, barley species and ecogeographic membership. The sampling was taken in the years 2015–2017.

| **Name** | **Species** | **Latitude** | **Longtitude** | **Cluster Membership** |
| --- | --- | --- | --- | --- |
| ALONEY HABASHAN | H.S | 33.04540278 | 35.8340472 | 1 |
| ALUMIM | H.S | 31.45102778 | 34.5173889 | 2 |
| BEIT DAGAN | H.V | 31.99410278 | 34.8190194 | 4 |
| EL H'HAR | H.G | 31.74788889 | 35.0445556 | 1 |
| GAN YAVNNE | H.S | 31.78433333 | 34.70325 | 4 |
| GAT V | H.S+H.V | 31.63005556 | 34.7985 | 2 |
| GILAT G | H.G | 31.33508333 | 34.6646667 | 2 |
| GIMZU | H.S | 31.95255556 | 34.94 | 5 |
| HARISH | H.S | 32.460628 | 35.048189 | 3 |
| HARUVIT FOREST | H.S | 31.72727778 | 34.8706389 | 5 |
| K.MASARIK | H.V | 32.89333333 | 35.1008333 | 3 |
| KATZIR | H.S | 32.484929 | 35.110856 | 3 |
| KISUFIM | H.S+H.V | 31.37805556 | 34.3833611 | 2 |
| MEYTZAR | H.S | 32.76644444 | 35.7337222 | 1 |
| MITZPE ILAN | H.S | 32.460279 | 35.069079 | 3 |
| MODIEIN | H.S | 31.87222222 | 35.0086111 | 5 |
| NETIV HA'LAMED HE | H.S | 31.67263889 | 35.0511389 | 1 |
| NAHAL RAZ | H.S | 32.57680556 | 35.0866111 | 3 |
| RISHON LETZION | H.S | 31.97693611 | 34.7885111 | 4 |
| SHTULIM | H.S+H.V | 31.78210556 | 34.6736944 | 4 |
| TIMRAT | H.S | 32.69905556 | 35.2141111 | 5 |
| TIVON | H.S | 32.75388889 | 35.1270556 | 3 |
| RAMOT MENASHE | H.S+H.V | 32.59836111 | 35.0634722 | 3 |
| ALMAGOR S | H.S | 32.91686111 | 35.6010833 | 6 |
| ALMAGOR S | H.S+H.B | 32.90327778 | 35.5998889 | 6 |
| ALONEY ABA | H.S | 32.733 | 35.1683333 | 3 |
| ALONEY KADIMA | H.S | 32.28852778 | 34.9282778 | 3 |
| AMIAD | H.S | 32.91333333 | 35.5431944 | 6 |
| BEERI | H.S+H.V | 31.42886111 | 34.4747778 | 2 |
| BEIT HANAN G | H.G | 31.93611111 | 34.7544444 | 4 |
| BEIT HANAN S | H.S | 31.93361111 | 34.7527778 | 4 |
| BEIT HASHITA | H.S | 32.54475 | 35.4311667 | 6 |
| BEIT KAMA | H.S+H.V | 31.45327778 | 34.7665556 | 2 |
| BEIT KESHET | H.S | 32.72361111 | 35.41 | 5 |
| BEIT SHEMESH | H.S | 31.75138889 | 35.0006944 | 5 |
| BIKAAT HASHITA | H.S | 32.55083333 | 35.4069444 | 6 |
| BINYAMINA | H.S | 32.51805556 | 34.9579167 | 3 |
| BIZARON | H.S | 31.79561111 | 34.7301389 | 4 |
| DALYA | H.S | 32.58775 | 35.0665556 | 3 |
| DAMOON | H.S+H.B | 32.89944444 | 35.1386111 | 3 |
| EYN HAEMEK | H.S | 32.63441667 | 35.0841667 | 3 |
| FAHEM | H.S | 32.38555556 | 35.1665278 | 3 |
| GAN YAOSHIYA | H.G | 32.34461111 | 34.9899444 | 3 |
| GAT B | H.S+H.V+H.G | 31.631 | 34.7978056 | 2 |
| GEFEN S | H.S | 31.74638889 | 34.8708611 | 5 |
| GEFEN V | H.V | 31.74638889 | 34.8708611 | 5 |
| GILAT V | H.V | 31.33580556 | 34.6649444 | 2 |
| GIVAT HAMORE | H.B | 32.61833333 | 35.3686111 | 5 |
| GIVAT HAMORE | H.S | 32.62055556 | 35.3372222 | 5 |
| GIVA'T HATURMUSIM | H.S | 32.55083333 | 35.4069444 | 6 |
| GONEN | H.S+H.V | 33.12097222 | 35.6421389 | 6 |
| HAD HALOM | H.S | 31.78061111 | 34.67025 | 4 |
| HAMAT GADER | H.S+H.G | 32.68479167 | 35.6670139 | 6 |
| HAR GIBORIM | H.S+H.G | 32.53208333 | 35.3641667 | 5 |
| HATAYASIM MOUNTAIN | H.G | 31.774175 | 35.09 | 1 |
| HAZOREA | H.S | 32.63441667 | 35.0841667 | 3 |
| K. HAROSHET | H.S | 32.69416667 | 35.1086111 | 3 |
| KAHAL | H.S+H.B | 32.88675 | 35.5100278 | 6 |
| KFAR DANIEL | H.S+H.G+H.B | 31.93375 | 34.93225 | 5 |
| KIRYAT TIVON-PESEL ZAID | H.G | 32.70116667 | 35.1278889 | 3 |
| LAHAV FOREST | H.S+H.G | 31.365 | 34.8505556 | 1 |
| MALAHIM FOREST | H.B | 31.59841667 | 34.8353611 | 2 |
| MAPALIM JUNCTION | H.S | 32.98611111 | 35.7503611 | 1 |
| MITZPE HAREL | H.S+H.B | 31.80200278 | 34.9604083 | 5 |
| MITZPE VINIA | H.S+H.G | 32.52472222 | 35.3875 | 5 |
| MODIEIN | H.G | 31.87658333 | 35.0096111 | 5 |
| MOSHAV PATISH | H.G | 31.33105556 | 34.5503333 | 2 |
| MOTZA EILIT | H.B | 31.79555556 | 35.1505556 | 1 |
| NAHAL ETZIONA | H.G | 31.67447222 | 35.0210833 | 5 |
| NAHAL GRAR | H.S+H.G | 31.37922222 | 34.6158889 | 2 |
| NAHAL KATLAV | H.S | 31.73677778 | 35.0785278 | 1 |
| NAHAL LAKISH | H.S+H.V | 31.77766667 | 34.6692222 | 4 |
| NAHAL SHIKMA | H.S | 31.48672222 | 34.7098611 | 2 |
| NETIVOT | H.S | 31.43252778 | 34.5851389 | 2 |
| NEVE MICHAEL | H.S | 31.670546 | 35.007188 | 5 |
| NIRIM | H.V | 31.3395 | 34.3836667 | 2 |
| PURA | H.B | 31.49538889 | 34.7761944 | 2 |
| RAMAT YOHANAN | H.V | 32.79852778 | 35.1249167 | 3 |
| RUHAMA | H.S | 31.48672222 | 34.7098611 | 2 |
| SHANI LIVNA | H.S+H.B | 31.35397222 | 35.0765083 | 1 |
| SHATA | H.S | 32.5485 | 35.4149167 | 6 |
| SUFA | H.S | 33.03502778 | 35.6908056 | 1 |
| TAL EL | H.S | 32.92511111 | 35.1725 | 3 |
| TAU | H.S | 32.11430556 | 34.8058056 | 4 |
| TEL ARAD | H.V | 31.25691667 | 35.1186528 | 1 |
| TIDHAR | H.S+H.B | 31.37922222 | 34.6158889 | 2 |
| TIMRAT | H.G+HB | 32.70111111 | 35.2152778 | 5 |
| TIVON | H.S | 32.70388889 | 35.1270556 | 3 |
| TIVON | H.G | 32.71186111 | 35.1522222 | 3 |
| TZOMET YHUDIYA | H.S | 32.90325 | 35.6470833 | 6 |
| YATIR FOREST | H.S+H.B | 31.34697222 | 35.0308056 | 1 |
| ZORAA FOREST-TARUM | H.S+H.B | 31.78383333 | 34.9775278 | 5 |

Marked examples, indicate sites that *ptt/ptm* isolate was isolated from the sample. The cluster membership is based on the ecogeographic characterises

**Table S2.** Summary of the mean value of the eco-geographic variable.

| **Temperature Annual Range** | **Temperature of Coldest Quarter** | **Precipitation of Coldest Quarter** | **Average Solar Radiation Jan-Mar** | **Average Wind January-March** | **N Rows** | **Eco-Geographic Group** |
| --- | --- | --- | --- | --- | --- | --- |
| 3.625 ± 0.026 *** | 1.495 ± 0.047 * | 1.05 ± 0.084 ** | 6.753 ± 0.089 ** | 2.160 ± 0.033 ** | 13 | 1 |
| 3.287 ± 0.033 * | 1.880 ± 0.008 ** | 0.726 ± 0.031 * | 7.156 ± 0.012 *** | 2.371 ± 0.043 *** | 17 | 2 |
| 3.248 ± 0.015 * | 1.848 ± 0.017 ** | 1.307 ± 0.025 *** | 6.452 ± 0.019 * | 2.373 ± 0.020 *** | 22 | 3 |
| 3.235 ± 0.013 * | 1.921 ± 0.01 *** | 1.265 ± 0.040 **/*** | 6.909 ± 0.026 ** | 2.536 ± 0.023 **** | 10 | 4 |
| 3.461 ± 0.013 ** | 1.825 ± 0.013 ** | 1.156 ± 0.026 **/*** | 6.756 ± 0.053 ** | 2.166 ± 0.026 ** | 19 | 5 |
| 3.772 ± 0.030 **** | 1.923 ± 0.01 ***1 | 1.048 ± 0.039 ** | 6.446 ± 0.034 * | 1.886 ± 0.032 * | 11 | 6 |

*, **, ***, and ****are represent significant differences between each group and tested in Wilcoxon method (P < 0.05))

**Table S3.** Aggressiveness of *Pyrenophora teres* isolates on detached leaves and saprophytic.

| **Isolate Name** | **Barke** | **Sagiv** | **Ma'anit** | **Noga** | **Saprophytic *** |
| --- | --- | --- | --- | --- | --- |
| HS-MA-TI | 0.399 ^A^ | 0.311 ^AB^ | 0.316 ^A^ | 0.295 ^A^ | 0.627 ^ABC^ |
| HS-MA-ME | 0.330 ^AB^ | 0.305 ^AB^ | 0.288 ^ABC^ | 0.265 ^AB^ | 0.667 ^ABC^ |
| HS-MA-RM | 0.292 ^ABC^ | 0.303 ^AB^ | 0.291 ^AB^ | 0.234 ^ABC^ | 0.582 ^ABC^ |
| HS-MA-NR | 0.329 ^AB^ | 0.282 ^ABC^ | 0.288 ^AB^ | 0.227 ^ABC^ | 0.589 ^ABC^ |
| HS-MA-GY | 0.286 ^ABC^ | 0.198 ^A BCDE^ | 0.214 ^ABCDEF^ | 0.208 ^ABCD^ | 0.581 ^ABC^ |
| HG-TE-EH | 0.196 ^ABCD^ | 0.189 ^A BCDE^ | 0.136 ^BCDEFGH^ | 0.207 ^ABCD^ | 0.580 ^ABC^ |
| HS-MA-RI | 0.310 ^ABC^ | 0.236 ^ABCD^ | 0.222 ^ABCDE^ | 0.194 ^ABCD^ | 0.559 ^ABC^ |
| HS-MA-NM | 0.306 ^ABC^ | 0.281 ^ABC^ | 0.241 ^ABCD^ | 0.183 ^ABCD^ | 0.680 ^AB^ |
| HV-TE-G A | 0.216 ^ABCD^ | 0.160 ^CDEF^ | 0.136 ^BCDEFGH^ | 0.165 ^ABCDE^ | 0.409 ^BCDE^ |
| HS-TE-MO | 0.189 ^ABCD^ | 0.141 ^CDEF^ | 0.088^FGHI^ | 0.164 ^ABCDE^ | 0.391 ^CDEF^ |
| HV-TE-NRM | 0.190 ^ABCD^ | 0.153 ^BCDEF^ | 0.085^GHI^ | 0.147 ^BCDE^ | 0.576 ^ABC^ |
| HV-MA-BD2 | 0.214 ^ABCD^ | 0.191 ^ABCDE^ | 0.132 ^ABCDEFGH^ | 0.140 ^BCDE^ | 0.575 ^ABC^ |
| HG-MA-GIL | 0.121 ^CD^ | 0.086 ^DEF^ | 0.093 ^DEFGHI^ | 0.138 ^BCDE^ | 0.413 ^BCDE^ |
| HS-TE-MI | 0.211 ^ABCD^ | 0.180 ^BCDEF^ | 0.122 ^DEFGH^ | 0.130 ^BCDE^ | 0.526 ^ABCD^ |
| HV-MA-KM | 0.195 ^ABCD^ | 0.183 ^ABCDE^ | 0.137 ^A BCDEFGH^ | 0.129 ^BCDE^ | - ** |
| HV-TE-ERM | 0.180 ^ABCD^ | 0.147 ^BCDEF^ | 0.086 ^EFGHI^ | 0.125 ^BCDE^ | 0.264 ^DEF^ |
| HS-TE-HAR | 0.208 ^ABCD^ | 0.387 ^A^ | 0.220 ^A BCDEFG^ | 0.123 ^BCDE^ | 0.502 ^ABCD^ |
| HS-TE-AL | 0.205 ^ABCD^ | 0.192 ^A BCDE^ | 0.115 ^CDEFGH^ | 0.122 ^BCDEF^ | 0.702 ^AB^ |
| HS-MA-KI | 0.176 ^ABCD^ | 0.109 ^CDEF^ | 0.081 ^GHI^ | 0.121 ^BCDEF^ | 0.606 ^ABC^ |
| HS-TE-KT | 0.177 ^ABCD^ | 0.125 ^BCDEF^ | 0.109 ^EFGH^ | 0.106 ^CDEF^ | 0.719 ^A^ |
| HS-TE-AH | 0.139 ^ABCD^ | 0.286 ^ABC^ | 0.128 ^ABCDEFGHI^ | 0.104 ^CDEF^ | 0.517 ^ABCD^ |
| HS-MA-GIM | 0.163 ^BCD^ | 0.127 ^BCDEF^ | 0.132 ^ABCDEFGH^ | 0.100 ^CDEF^ | 0.454 ^A BCDE^ |
| HV-MA-BD1 | 0.166 ^BCD^ | 0.129 ^BCDEF^ | 0.113 ^DEFGH^ | 0.079 ^DEF^ | 0.598 ^ABC^ |
| HS-TE-KA | 0.167 ^BCD^ | 0.130 ^BCDEF^ | 0.077 ^GHI^ | 0.077 ^DEF^ | 0.658 ^ABC^ |
| HS-TE-SH | 0.110 ^CD^ | 0.076 ^EF^ | 0.079 ^GHI^ | 0.052 ^EF^ | 0.124 ^F^ |
| HS-TE-NH | 0.119 ^CD^ | 0.059 ^EF^ | 0.046 ^HI^ | 0.04 ^EF^ | 0.550 ^ABCD^ |
| HS-TE-HA | 0.056 ^D^ | 0.032 ^F^ | 0.028 ^I^ | 0.015 ^F^ | 0.180 ^EF^ |

Different letters differ significAntly, as determined by Tukey’s highly significant difference test, at *P* < 0.05. *Correlation between rate of progress on detached leaves and saprophytic medium were tested by Pearson correlation coefficient and found significant (r=0.681, P < 0.0001). **The isolates showed high difference between the replicate due differences in freshness of the agar plate.
