# Supplementary material for: Characterization of the Barley Net Blotch Pathosystem at the Center of Origin of Host and Pathogen
Source: Pathogens. 2019 Nov 29;8(4):275. doi: 10.3390/pathogens8040275 (PMC6963742; doi:10.3390/pathogens8040275)
Supplement: Supplementary file 1 [file pathogens-08-00275-s001.zip › pathogens-649293-supplementary/supplementary.pdf]

Table S1. Distribution of Net Blotch disease in Israel by site, barley species and ecogeographic membership. The sampling was taken in the years 2015–2017.

Name	Species	Latitude	Longtitude	Cluster Membership
ALONEY HABASHAN	H.S	33.04540278	35.8340472	1
ALUMIM	H.S	31.45102778	34.5173889	2
BEIT DAGAN	H.V	31.99410278	34.8190194	4
EL H'HAR	H.G	31.74788889	35.0445556	1
GAN YAVNNE	H.S	31.78433333	34.70325	4
GAT V	H.S+H.V	31.63005556	34.7985	2
GILAT G	H.G	31.33508333	34.6646667	2
GIMZU	H.S	31.95255556	34.94	5
HARISH	H.S	32.460628	35.048189	3
HARUVIT FOREST	H.S	31.72727778	34.8706389	5
K.MASARIK	H.V	32.89333333	35.1008333	3
KATZIR	H.S	32.484929	35.110856	3
KISUFIM	H.S+H.V	31.37805556	34.3833611	2
MEYTZAR	H.S	32.76644444	35.7337222	1
MITZPE ILAN	H.S	32.460279	35.069079	3
MODIEIN	H.S	31.87222222	35.0086111	5
NETIV HA'LAMED HE	H.S	31.67263889	35.0511389	1
NAHAL RAZ	H.S	32.57680556	35.0866111	3
RISHON LETZION	H.S	31.97693611	34.7885111	4
SHTULIM	H.S+H.V	31.78210556	34.6736944	4
TIMRAT	H.S	32.69905556	35.2141111	5
TIVON	H.S	32.75388889	35.1270556	3
RAMOT MENASHE	H.S+H.V	32.59836111	35.0634722	3
ALMAGOR S	H.S	32.91686111	35.6010833	6
ALMAGOR S	H.S+H.B	32.90327778	35.5998889	6
ALONEY ABA	H.S	32.733	35.1683333	3
ALONEY KADIMA	H.S	32.28852778	34.9282778	3
AMIAD	H.S	32.91333333	35.5431944	6
BEERI	H.S+H.V	31.42886111	34.4747778	2
BEIT HANAN G	H.G	31.93611111	34.7544444	4
BEIT HANAN S	H.S	31.93361111	34.7527778	4
BEIT HASHITA	H.S	32.54475	35.4311667	6
BEIT KAMA	H.S+H.V	31.45327778	34.7665556	2
BEIT KESHET	H.S	32.72361111	35.41	5
BEIT SHEMESH	H.S	31.75138889	35.0006944	5
BIKAAT HASHITA	H.S	32.55083333	35.4069444	6
BINYAMINA	H.S	32.51805556	34.9579167	3
BIZARON	H.S	31.79561111	34.7301389	4
DALYA	H.S	32.58775	35.0665556	3
DAMOON	H.S+H.B	32.89944444	35.1386111	3
EYN HAEMEK	H.S	32.63441667	35.0841667	3
FAHEM	H.S	32.38555556	35.1665278	3
GAN YAOSHIYA	H.G	32.34461111	34.9899444	3
GAT B	H.S+H.V+H.G	31.631	34.7978056	2
GEFEN S	H.S	31.74638889	34.8708611	5
GEFEN V	H.V	31.74638889	34.8708611	5
GILAT V	H.V	31.33580556	34.6649444	2
GIVAT HAMORE	H.B	32.61833333	35.3686111	5
GIVAT HAMORE	H.S	32.62055556	35.3372222	5
GIVA'T HATURMUSIM	H.S	32.55083333	35.4069444	6
GONEN	H.S+H.V	33.12097222	35.6421389	6
HAD HALOM	H.S	31.78061111	34.67025	4
HAMAT GADER	H.S+H.G	32.68479167	35.6670139	6

HAR GIBORIM	H.S+H.G	32.53208333	35.3641667	5
HATAYASIM MOUNTAIN	H.G	31.774175	35.09	1
HAZOREA	H.S	32.63441667	35.0841667	3
K. HAROSHET	H.S	32.69416667	35.1086111	3
KAHAL	H.S+H.B	32.88675	35.5100278	6
KFAR DANIEL	H.S+H.G+H.B	31.93375	34.93225	5
KIRYAT TIVON-PESEL ZAID	H.G	32.70116667	35.1278889	3
LAHAV FOREST	H.S+H.G	31.365	34.8505556	1
MALAHIM FOREST	H.B	31.59841667	34.8353611	2
MAPALIM JUNCTION	H.S	32.98611111	35.7503611	1
MITZPE HAREL	H.S+H.B	31.80200278	34.9604083	5
MITZPE VINIA	H.S+H.G	32.52472222	35.3875	5
MODIEIN	H.G	31.87658333	35.0096111	5
MOSHAV PATISH	H.G	31.33105556	34.5503333	2
MOTZA EILIT	H.B	31.79555556	35.1505556	1
NAHAL ETZIONA	H.G	31.67447222	35.0210833	5
NAHAL GRAR	H.S+H.G	31.37922222	34.6158889	2
NAHAL KATLAV	H.S	31.73677778	35.0785278	1
NAHAL LAKISH	H.S+H.V	31.77766667	34.6692222	4
NAHAL SHIKMA	H.S	31.48672222	34.7098611	2
NETIVOT	H.S	31.43252778	34.5851389	2
NEVE MICHAEL	H.S	31.670546	35.007188	5
NIRIM	H.V	31.3395	34.3836667	2
PURA	H.B	31.49538889	34.7761944	2
RAMAT YOHANAN	H.V	32.79852778	35.1249167	3
RUHAMA	H.S	31.48672222	34.7098611	2
SHANI LIVNA	H.S+H.B	31.35397222	35.0765083	1
SHATA	H.S	32.5485	35.4149167	6
SUFA	H.S	33.03502778	35.6908056	1
TAL EL	H.S	32.92511111	35.1725	3
TAU	H.S	32.11430556	34.8058056	4
TEL ARAD	H.V	31.25691667	35.1186528	1
TIDHAR	H.S+H.B	31.37922222	34.6158889	2
TIMRAT	H.G+HB	32.70111111	35.2152778	5
TIVON	H.S	32.70388889	35.1270556	3
TIVON	H.G	32.71186111	35.1522222	3
TZOMET YHUDIYA	H.S	32.90325	35.6470833	6
YATIR FOREST	H.S+H.B	31.34697222	35.0308056	1
ZORAA FOREST-TARUM	H.S+H.B	31.78383333	34.9775278	5

Marked examples, indicate sites that *ptt/ptm* isolate was isolated from the sample. The cluster membership is based on the ecogeographic characterises

Table S2. Summary of the mean value of the eco-geographic variable.

Eco-Geographic Group	N Rows	Average Wind January-March	Average Solar Radiation Jan-Mar	Precipitation of Coldest Quarter	Temperature of Coldest Quarter	Temperature Annual Range
1	13	2.160 ± 0.033 **	6.753 ± 0.089 **	1.05 ± 0.084 **	1.495 ± 0.047 *	3.625 ± 0.026 ***
2	17	2.371 ± 0.043 ***	7.156 ± 0.012 ***	0.726 ± 0.031 *	1.880 ± 0.008 **	3.287 ± 0.033 *
3	22	2.373 ± 0.020 ***	6.452 ± 0.019 *	1.307 ± 0.025 ***	1.848 ± 0.017 **	3.248 ± 0.015 *
4	10	2.536 ± 0.023 ****	6.909 ± 0.026 **	1.265 ± 0.040 **/***	1.921 ± 0.01 ***	3.235 ± 0.013 *

5	19	2.166 ± 0.026 **	6.756 ± 0.053 **	1.156 ± 0.026 **/***	1.825 ± 0.013 **	3.461 ± 0.013 **
6	11	1.886 ± 0.032 *	6.446 ± 0.034 *	1.048 ± 0.039 **	1.923 ± 0.01 ***1	3.772 ± 0.030 ****

*, **, ***, and ****are represent significant differences between each group and tested in Wilcoxon method (P < 0.05))

Table S3. Aggressiveness of *Pyrenophora teres* isolates on detached leaves and saprophytic.

Isolate Name	Barke	Sagiv	Ma'anit	Noga	Saprophytic *
HS-MA-TI	0.399 ^{A}	0.311 ^{AB}	0.316 ^{A}	0.295 ^{A}	0.627 ^{ABC}
HS-MA-ME	0.330 ^{AB}	0.305 ^{AB}	0.288 ^{ABC}	0.265 ^{AB}	0.667 ^{ABC}
HS-MA-RM	0.292 ^{ABC}	0.303 ^{AB}	0.291 ^{AB}	0.234 ^{ABC}	0.582 ^{ABC}
HS-MA-NR	0.329 ^{AB}	0.282 ^{ABC}	0.288 ^{AB}	0.227 ^{ABC}	0.589 ^{ABC}
HS-MA-GY	0.286 ^{ABC}	0.198 $^{A\ BCDE}$	0.214 ^{ABCDEF}	0.208 ^{ABCD}	0.581 ^{ABC}
HG-TE-EH	0.196 ^{ABCD}	0.189 $^{A\ BCDE}$	0.136 ^{BCDEFGH}	0.207 ^{ABCD}	0.580 ^{ABC}
HS-MA-RI	0.310 ^{ABC}	0.236 ^{ABCD}	0.222 ^{ABCDE}	0.194 ^{ABCD}	0.559 ^{ABC}
HS-MA-NM	0.306 ^{ABC}	0.281 ^{ABC}	0.241 ^{ABCD}	0.183 ^{ABCD}	0.680 ^{AB}
HV-TE-G A	0.216 ^{ABCD}	0.160 ^{CDEF}	0.136 ^{BCDEFGH}	0.165 ^{ABCDE}	0.409 ^{BCDE}
HS-TE-MO	0.189 ^{ABCD}	0.141 ^{CDEF}	0.088 ^{FGHI}	0.164 ^{ABCDE}	0.391 ^{CDEF}
HV-TE-NRM	0.190 ^{ABCD}	0.153 ^{BCDEF}	0.085 ^{GHI}	0.147 ^{BCDE}	0.576 ^{ABC}
HV-MA-BD2	0.214 ^{ABCD}	0.191 ^{ABCDE}	0.132 ^{ABCDEFGH}	0.140 ^{BCDE}	0.575 ^{ABC}
HG-MA-GIL	0.121 ^{CD}	0.086 ^{DEF}	0.093 ^{DEFGHI}	0.138 ^{BCDE}	0.413 ^{BCDE}
HS-TE-MI	0.211 ^{ABCD}	0.180 ^{BCDEF}	0.122 ^{DEFGH}	0.130 ^{BCDE}	0.526 ^{ABCD}
HV-MA-KM	0.195 ^{ABCD}	0.183 ^{ABCDE}	0.137 $^{A\ BCDEFGH}$	0.129 ^{BCDE}	- **
HV-TE-ERM	0.180 ^{ABCD}	0.147 ^{BCDEF}	0.086 ^{EFGHI}	0.125 ^{BCDE}	0.264 ^{DEF}
HS-TE-HAR	0.208 ^{ABCD}	0.387 ^{A}	0.220 $^{A\ BCDEFG}$	0.123 ^{BCDE}	0.502 ^{ABCD}
HS-TE-AL	0.205 ^{ABCD}	0.192 $^{A\ BCDE}$	0.115 ^{CDEFGH}	0.122 ^{BCDEF}	0.702 ^{AB}
HS-MA-KI	0.176 ^{ABCD}	0.109 ^{CDEF}	0.081 ^{GHI}	0.121 ^{BCDEF}	0.606 ^{ABC}
HS-TE-KT	0.177 ^{ABCD}	0.125 ^{BCDEF}	0.109 ^{EFGH}	0.106 ^{CDEF}	0.719 ^{A}
HS-TE-AH	0.139 ^{ABCD}	0.286 ^{ABC}	0.128 ^{ABCDEFGHI}	0.104 ^{CDEF}	0.517 ^{ABCD}
HS-MA-GIM	0.163 ^{BCD}	0.127 ^{BCDEF}	0.132 ^{ABCDEFGH}	0.100 ^{CDEF}	0.454 $^{A\ BCDE}$
HV-MA-BD1	0.166 ^{BCD}	0.129 ^{BCDEF}	0.113 ^{DEFGH}	0.079 ^{DEF}	0.598 ^{ABC}
HS-TE-KA	0.167 ^{BCD}	0.130 ^{BCDEF}	0.077 ^{GHI}	0.077 ^{DEF}	0.658 ^{ABC}
HS-TE-SH	0.110 ^{CD}	0.076 ^{EF}	0.079 ^{GHI}	0.052 ^{EF}	0.124 ^{F}
HS-TE-NH	0.119 ^{CD}	0.059 ^{EF}	0.046 ^{HI}	0.04 ^{EF}	0.550 ^{ABCD}
HS-TE-HA	0.056 ^{D}	0.032 ^{F}	0.028 ^{I}	0.015 ^{F}	0.180 ^{EF}

Different letters differ significAntly, as determined by Tukey’s highly significant difference test, at $P < 0.05$. *Correlation between rate of progress on detached leaves and saprophytic medium were tested by Pearson correlation coefficient and found significant (r=0.681, P < 0.0001). **The isolates showed high difference between the replicate due differences in freshness of the agar plate.
