# Supplementary figures and images for: Characterization of the Barley Net Blotch Pathosystem at the Center of Origin of Host and Pathogen
Source: Pathogens. 2019 Nov 29;8(4):275. doi: 10.3390/pathogens8040275 (PMC6963742; doi:10.3390/pathogens8040275)

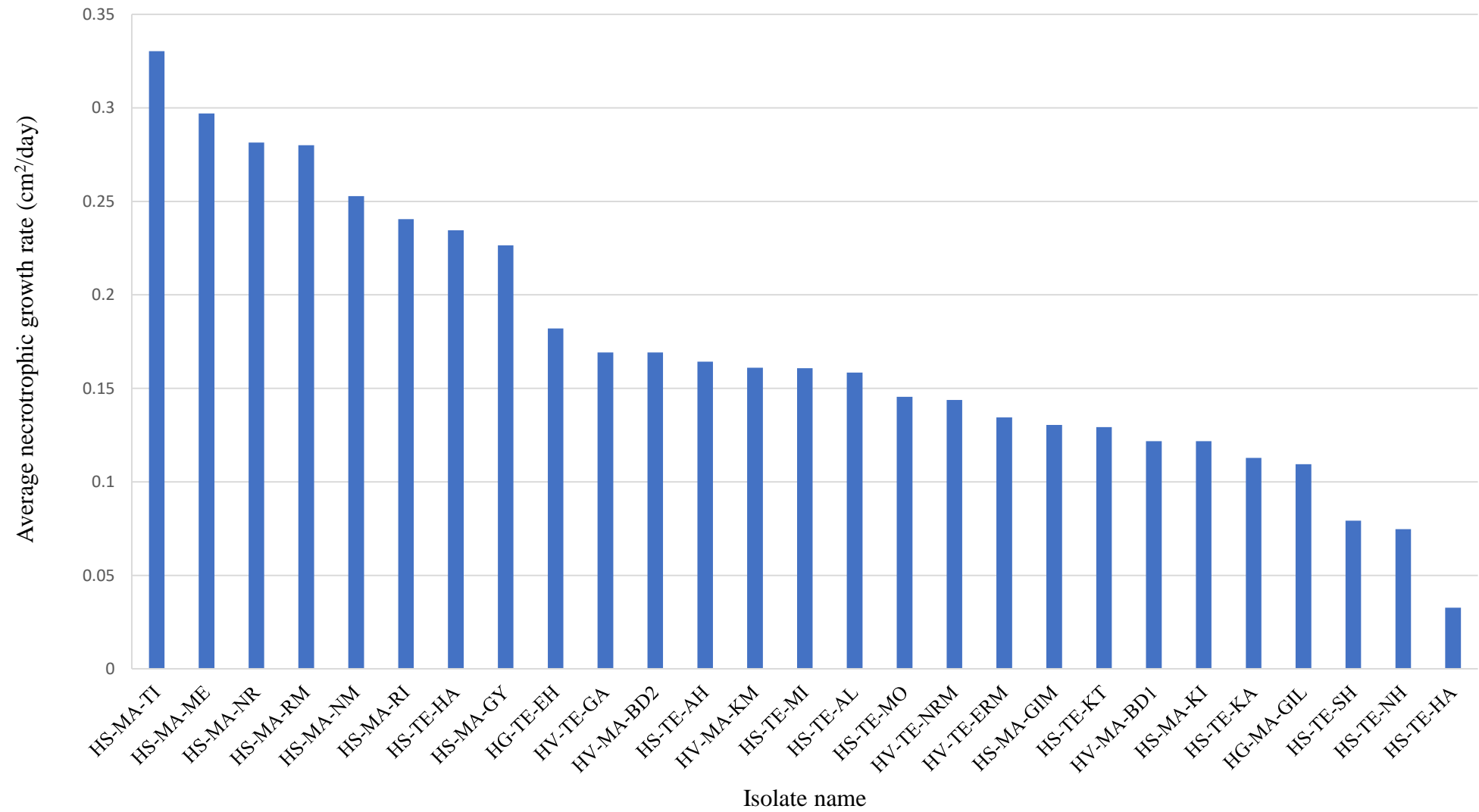

Supplement: Supplementary file 1 [file pathogens-08-00275-s001.zip › pathogens-649293-supplementary/figure s1.pdf]
